# 10th Anniversary of Biomedicines—Advances in Mesenchymal Stem Cells

**DOI:** 10.3390/biomedicines11082183

**Published:** 2023-08-03

**Authors:** Vincenzo Mattei, Simona Delle Monache

**Affiliations:** 1 Biomedicine and Advanced Technologies Rieti Center, Sabina Universitas, 02100 Rieti, Italy; 2 Department of Biotechnological and Applied Clinical Sciences, University of L’Aquila, 67100 L’Aquila, Italy

Mesenchymal stromal cells (MSCs) are non-specialized adult stem cells (ASCs), cells that reproduce to provide specific cytotypes [1]. MSCs were first recognized and identified in bone marrow in the 1970s [2]. To date, other MSC sources have also been identified, such as umbilical cord, amniotic fluid, subcutis, dental tissue, skeletal muscle, synovium, liver, lungs, and placenta [3,4,5,6,7,8]. In 2006, the Mesenchymal and Tissue Stem Cell Committee of the International Society for Cellular Therapy proposed three minimal properties for defining human MSCs: plastic adherence, multilineage differentiation (chondrocytes, osteoblasts, and adipocytes), and the expression of specific markers (CD105, CD73, and CD90) [9]. MSCs and their conditioned media (CM) are considered to be promising in regenerative–reparative medicine, cell therapy, and tissue engineering [10,11,12,13,14] due to their effects on tissue and organ homeostasis, revascularization, and tissue repair, as well as their immunomodulatory, anti-inflammatory, pro-angiogenic, pleiotropic, and tropic abilities. For this reason, considerable efforts have been made to introducing advanced MSC-based therapy into clinical practice. This Special Issue seeks to collect the latest innovative findings and achievements in the field of MSC-based therapy. Sarikidi et al. discuss the potential role of the immunomodulatory mechanism of mesenchymal stem cells in amyotrophic lateral sclerosis (ALS) [15]. The authors investigated the effects of hMSCs and an hMSC-CM on Cu/Zn superoxidase dismutase 1G93A (SOD1G93A) transgenic primary motor neurons (MNs). They showed that the co-culture of hMSCs and MNs resulted in slightly higher MN numbers, but did not protect against staurosporine (STS)-induced toxicity, implying marginal direct trophic effects of hMSCs. They found high levels of vascular endothelial growth factor (VEGF) and C-X3-C motif chemokine 1 (CX3CL1) in the hMSC secretome. Moreover, the co-culture of hMSCs and MNs showed an altered gene expression of growth factors and cytokines/chemokines in both MNs and hMSCs. In addition, hMSCs cocultured with SOD1G93A MNs showed the upregulation of CX3CL1 and its receptor CX3CR1 and the downregulation of interleukin- 1 β (IL1β) and interleukin-8 (IL8). Conversely, MNs exhibited the upregulation of some growth factors as well as CX3CR1 upon hMSC co-culture. The authors indicated that hMSCs only provide moderate trophic support to MNs via growth factor gene regulation and may mediate anti-inflammatory responses through the CX3CL1/CX3CR1 axis, but also increase the expression of pro-inflammatory cytokines, which limits their therapeutic potential.

Santilli et al. discuss the role of gangliosides (GGs) in the multilineage differentiation process of mesenchymal stem cells of several types, such as umbilical cord-derived MSCs (UC-MSCs), bone marrow-derived MSCs (BM-MSCs), dental pulp-derived MSCs (DPSCs), and adipose-derived MSCs (ADSCs). They evidenced a role of GGs as specific markers of the cellular state of undifferentiated and differentiated MSCs that are involved in the multilineage differentiation process (osteogenic, chondrogenic, neurogenic, and adipogenic lineage). Moreover, they examined the possible role of GGs as specific cell surface markers to identify or isolate specific mesenchymal stem cell isotypes and their potential use as additional markers for the quality control of cell-based therapies [16]. Several authors showed a switch in the ganglioside pattern during the multilineage differentiation process of different MSCs [17,18,19,20]. Moreover, the identification of the expression of specific GGs during a different stage of MSC commitment may enable the use of these molecules as potential targets to isolate specific MSC clones (magnetic immunodetection). The authors conclude that a better knowledge of ganglioside expression in stem cells will allow for the better separation of specific clones for their utilization in regenerative medicine. Zhu et al., in their paper, aimed to examine the efficacy of hypoxia-inducible factor (HIF)–1α-overexpressing MSC (HIF-MSC) transplantation in experimental colitis and investigated the immunity regulation mechanisms of HIF-MSC via macrophages. They showed that HIF-MSC transplantation significantly attenuated colitis in terms of weight loss rate, disease activity index (DAI), colon length, and pathology score, and effectively rebuilt the local and systemic immune balance. Moreover, they observed that HIF-MSCs significantly decreased the number of M1-like macrophages and increased the number of M2-like macrophages in colon tissues. Moreover, co-culture with HIF-MSCs reduces the expression of pro-inflammatory factors (CCR-7 and inducible nitric oxide synthase) and increased the expression of anti-inflammatory factors and arginase I in induced M1-like macrophages. In addition, they observed the upregulation of the expression of downstream molecular targets of phosphatidylinositol 3-kinase-γ (HIF-1α, p-AKT/AKT) in the colon tissue during HIF-MSC treatment. Finally, the authors demonstrated that modified MSCs over-expressed HIF-1α, which effectively regulated macrophage polarization through PI3K-γ. In conclusion, the authors showed that HIF-MSC transplantation in mice with experimental colitis resulted in a good immune balance and mucosal rehabilitation, thereby proving to be a potentially effective treatment for IBD or other inflammatory diseases [21]. Several authors have shown that the MSC secretome contains the anti-inflammatory cytokines IL-10 and TGF-β, along with other uncharacterized molecules [22]. In this view, Paganelli et al. discuss the role of Galectin-3 (GAL-3), a molecule produced by MSCs and other cell sources under inflammatory conditions. In fact, GAL-3 seems to be highly expressed by MSCs under inflammatory conditions [23], is exposed on the cellular membrane surface, and is released in soluble form. Based on previous findings reporting an anti-inflammatory action mediated by its natural ligand GAL-3BP, in their paper, the authors evaluated the regulation of GAL-3 in LPS-induced inflammation in human gingival mesenchymal stem cells (hGMSCs). hGMSCs were stimulated with LPSs, and the expression of NFκB p65, MyD88, and NALP3 were evaluated with or without GAL-3, utilizing immunofluorescence, Western blot assay, and RT- PCR. They showed that GAL-3 inhibited the expression of TLR4, NFκB p65, MyD88, and NALP3 in hGMSCs induced via LPSs. Moreover, the addition of either GAL3-BP or the antibody to GAL-3 blocks GAL-3-mediated effects and restores the expression of LR4, NFκB p65, MyD88, and NALP3. The authors conclude that GAL-3 induces the downregulation of the LPS-induced inflammatory program in MSCs and modulates the LPS-induced inflammatory program in MSCs.

In this Special Issue, some papers have been collected in which the potential role of MSCs and their secretome on tissue and organ homeostasis, revascularization, and tissue repair, as well as their immunomodulatory and anti-inflammatory capacities, have been investigated. This role seems to be different depending on the selected cytotype, providing, in some cases, trophic support with a rebalancing of the immune system, and sometimes an increase in proinflammatory cytokines, limiting, in this case, their utilization in regenerative medicine. Therefore, the need emerges for greater knowledge and characterization of the different cytotypes of MSCs which could be based on the expression of specific gangliosides, which would thus represent real markers of the specific MSC cytotype.

## References

[1] Hass R., Kasper C., Böhm S., Jacobs R. (2011). Different populations and sources of human mesenchymal stem cells (MSC): A comparison of adult and neonatal tissue derived MSC. Cell Commun. Signal..

[2] Friedenstein A.J., Chailakhjan R.K., Lalykina K.S. (1970). The development of fibroblast colonies in monolayer cultures of guinea-pig bone marrow and spleen cells. Cell Tissue Kinet..

[3] Can A., Karahuseyinoglu S. (2007). Concise review: Human umbilical cord stroma with regard to the source of fetus-derived stem cells. Stem. Cells.

[4] Huang J., Ma W., Wei X., Yuan Z. (2020). Amniotic fluid mesenchymal stromal cells from early stages of embryonic development have higher self-renewal potential. Vitr. Cell Dev. Biol. Anim..

[5] Shin S., El-Sabbagh A.S., Lukas B.E., Tanneberger S.J., Jiang Y. (2020). Adipose stem cells in obesity: Challenges and opportunities. Biosci. Rep..

[6] Gronthos S., Mankani M., Brahim J., Robey P.G., Shi S. (2000). Postnatal human dental pulp stem cells (DPSCs) in vitro and in vivo. Proc. Natl. Acad. Sci. USA.

[7] Delle Monache S., Pulcini F., Frosini R., Mattei V., Talesa V.N., Antognelli C. (2021). Methylglyoxal-Dependent Glycative Stress Is Prevented by the Natural Antioxidant Oleuropein in Human Dental Pulp Stem Cells through Nrf2/Glo1 Pathway. Antioxidants.

[8] Mushahary D., Spittler A., Kasper C., Weber V., Charwat V. (2018). Isolation, cultivation, and characterization of human mesenchymal stem cells. Cytom. A.

[9] Dominici M., Le Blanc K., Mueller I., Slaper-Cortenbach I., Marini F., Krause D., Deans R., Keating A., Prockop D., Horwitz E. (2006). Minimal criteria for defining multipotent mesenchymal stromal cells. The International Society for Cellular Therapy position statement. Cytotherapy.

[10] Martellucci S., Santacroce C., Manganelli V., Santilli F., Piccoli L., Cassetta M., Misasi R., Sorice M., Mattei V. (2019). Isolation, Propagation, and Prion Protein Expression During Neuronal Differentiation of Human Dental Pulp Stem Cells. J. Vis. Exp..

[11] Vizoso F.J., Eiro N., Cid S., Schneider J., Perez-Fernandez R. (2017). Mesenchymal Stem Cell Secretome: Toward Cell-Free Therapeutic Strategies in Regenerative Medicine. Int. J. Mol. Sci..

[12] Prakash N., Kim J., Jeon J., Kim S., Arai Y., Bello A.B., Park H., Lee S.H. (2023). Progress and emerging techniques for biomaterial-based derivation of mesenchymal stem cells (MSCs) from pluripotent stem cells (PSCs). Biomater. Res..

[13] Delle Monache S., Pulcini F., Santilli F., Martellucci S., Santacroce C., Fabrizi J., Angelucci A., Sorice M., Mattei V. (2022). Hypoxia Induces DPSC Differentiation versus a Neurogenic Phenotype by the Paracrine Mechanism. Biomedicines.

[14] Candelise N., Santilli F., Fabrizi J., Caissutti D., Spinello Z., Moliterni C., Lancia L., Delle Monache S., Mattei V., Misasi R. (2023). The Importance of Stem Cells Isolated from Human Dental Pulp and Exfoliated Deciduous Teeth as Therapeutic Approach in Nervous System Pathologies. Cells.

[15] Rufino R.A., Pereira-Rufino L.D.S., Vissoto T.C.S., Kerkis I., Neves A.D.C., da Silva M.C.P. (2022). The Immunomodulatory Potential Role of Mesenchymal Stem Cells in Diseases of the Central Nervous System. Neurodegener. Dis..

[16] Hohenwallner K., Troppmair N., Panzenboeck L., Kasper C., El Abiead Y., Koellensperger G., Lamp L.M., Hartler J., Egger D., Rampler E. (2022). Decoding distinct ganglioside patterns of native and differentiated mesenchymal stem cells by a novel glycolipidomics profiling strategy. JACS Au.

[17] Ryu J.S., Ko K., Lee J.W., Park S.B., Byun S.J., Jeong E.J., Ko K., Choo Y.K. (2009). Gangliosides are involved in neural differentiation of human dental pulp-derived stem cells. Biochem. Biophys. Res. Commun..

[18] Kim S.M., Jung J.U., Ryu J.S., Jin J.W., Yang H.J., Ko K., You H.K., Jung K.Y., Choo Y.K. (2008). Effects of gangliosides on the differentiation of human mesenchymal stem cells into osteoblasts by modulating epidermal growth factor receptors. Biochem. Biophys. Res. Commun..

[19] Nan C., Shi Y., Zhao Z., Ma S., Liu J., Yan D., Song G., Liu H. (2015). Monosialoteterahexosyl ganglioside induces the differentiation of human umbilical cord-derived mesenchymal stem cells into neuron-like cells. Int. J. Mol. Med..

[20] Martellucci S., Manganelli V., Santacroce C., Santilli F., Piccoli L., Sorice M., Mattei V. (2018). Role of Prion protein-EGFR multimolecular complex during neuronal differentiation of human dental pulp-derived stem cells. Prion.

[21] Neurath M.F. (2017). Current and emerging therapeutic targets for IBD. Nat. Rev. Gastroenterol. Hepatol..

[22] Xia T.T., Hu R., Shao C.J., Feng Y., Yang X.L., Xie Y.P., Shi J.X., Li J.S., Li X.M. (2023). Stanniocalcin-1 secreted by human umbilical mesenchymal stem cells regulates interleukin-10 expression via the PI3K/AKT/mTOR pathway in alveolar macrophages. Cytokine.

[23] Soares L.C., Al-Dalahmah O., Hillis J., Young C.C., Asbed I., Sakaguchi M., O’Neill E., Szele F.G. (2021). Novel Galectin-3 Roles in Neurogenesis, Inflammation and Neurological Diseases. Cells.

